# Gender differences in self-assessed performance and stress level during training of basic interventional radiology maneuvers

**DOI:** 10.1007/s00330-023-09993-3

**Published:** 2023-08-08

**Authors:** Sebastian R. Reder, Annaig Rohou, Naureen Keric, Katja U. Beiser, Ahmed E. Othman, Mario Alberto Abello Mercado, Sebastian Altmann, Katja Petrowski, Marc A. Brockmann, Carolin Brockmann

**Affiliations:** 1 Department of Neuroradiology, University Medical Centre of the Johannes Gutenberg-University, Langenbeckstr. 1, Mainz, 55131 Germany; 2 Faculty of Psychology, University of Graz, Graz, Austria; 3 Department of Neurosurgery, University Medical Centre of the Johannes Gutenberg-University, Langenbeckstr. 1, Mainz, 55131, Germany; 4 Department of Medical Psychology and Medical Sociology, University Medical Centre of the Johannes Gutenberg-University, Duesbergweg 6, Mainz, 55128 Germany

**Keywords:** Gender differences, Self-assessment, Interventional radiology, Educational measurement

## Abstract

**Objectives:**

Gender differences have been reported to influence medical training. We investigated gender differences encountered during training in interventional radiology maneuvers.

**Methods:**

Catheter handling was analyzed under standardized conditions in 64 participants naïve to endovascular procedures (26 women, 38 men). Objective (e.g., catheter pathway, catheter movements, required time) and subjective parameters (stress level) were recorded. The NASA-Task Load Index (NASA-TLX; 1–20 points) was used to assess participants’ stress levels and perceived workload.

**Results:**

In the easier tasks, no significant differences between male and female participants regarding catheter handling were observed. In the most complex task, female participants took themselves more time (688 ± 363 vs. 501 ± 230 s; *p *= 0.02), asked for help more frequently (*n *= 19 vs. *n *= 8) and earlier than men (203 ± 94 vs. 305 ± 142 s; *p *= 0.049), whereas men stood out by more agitated catheter handling (6.0 ± 1.8 vs. 4.8 ± 1.6 movements/s; *p *= 0.005). Overall, female participants perceived tasks to be more difficult (11.5 ± 4.2 vs. 9.6 ± 3.3; *p *= 0.016), perceived higher stress levels (8.9 ± 4.9 vs. 6.3 ± 4.4; *p *= 0.037), and rated their own performance lower (9.12 ± 3.3 vs. 11.3 ± 3.3; *p *= 0.009). However, female participants were able to correlate self-assessed with objective parameters correctly (*r* between −0.555 and −0.469; *p *= 0.004–0.018), whereas male participants failed to correctly rate their performance (*p* between 0.34 and 0.73). Stress levels correlated with objective parameters in males (*r* between 0.4 and 0.587; *p *< 0.005), but not in female participants.

**Conclusion:**

Perceived stress levels, self-evaluation skills, and catheter handling differ greatly between untrained male and female participants trying to solve interventional radiological tasks. These gender-specific differences should be considered in interventional radiology training.

**Clinical relevance statement:**

As psychological aspects may influence individual working strategies gender-specific differences in self-perception while learning interventional radiology maneuvers could be essential regarding success in teaching and treatment outcomes.

**Key Points:**

*• After performing standardized training, 38 male and 26 female volunteers showed significant differences regarding objective and self-assessed performance, as well as in perceived workload while performing simulated endovascular catheter maneuvers.*

*• After solving simulated endovascular radiological tasks, female participants were able to self-assess their objective performance much more accurately than male participants.*

*• Women took more time to solve simulated endovascular tasks and asked earlier and more frequently for help than men.*

## Introduction

Gender differences are known to influence physician-patient interaction and patient satisfaction [1–5], medical practice, and therapy outcomes [1, 2, 4–6]. In the case of objectively comparable medical performance, for example, patients rated treatment success depending on the doctor’s gender, which resulted in differences in receiving appreciation and positive public representation [3, 7–10]. Likewise, gender differences in medical specialty preferences have been described [11, 12].

Focusing on the broad field of radiology, less women working in the field of interventional radiology (IR) can be found [9, 10, 13]. Accordingly, Li et al also reported a significant gender disparity in IR compared to general radiology in Canada [14], which they attributed to possible misconceptions about IR among medical students and female physicians, presumably due to insufficient mentoring. However, they also mentioned pregnancy-related issues and gender differences in teamwork to contribute to these findings [14].

As various gender-related differences have been reported for medical education, it is imperative to discern their origin and the processes involved in their emergence to improve understanding and to take these disparities into consideration in the training of interventionalists. The underlying study therefore focuses on self-assessment in learning basic interventional endovascular tasks. Psychological variables, such as perceived stress levels and self-assessed ratings of performance and difficulty of different tasks were included in the paradigm to grant a differentiated understanding of the subjective processes and experiences that lead to these gender differences.

## Material and methods

### Participants, inclusion, and exclusion criteria

Sixty-four participants (26 female and 38 male) naïve to endovascular, interventional techniques were recruited from students and staff of University Medical Center Mainz always by the same male MD-student. There was no significant mediator effect within the study population characteristics (Table 1). To minimize a potential bias during recruitment, participants were recruited by hanging flyers or by asking a complete team before or after the case demonstration if any volunteers would like to take part in the underlying study. Three female and one male participant included in the study were known personally by the recruiter. One female participant (hitherto unknown to the recruiting MD student) quit during the second task as she perceived the difficulty of the task to be too high. Data was anonymized after collection. Participants were informed about data protection policy and gave informed consent. The research paradigm was non-invasive and posed no biological risk to participants. As participation was on a voluntary basis and study data were published anonymously, the Institutional Review Board waived issuing a statement. The study was conducted in accordance with the ethical standards as laid down in the 1964 Declaration of Helsinki and its later amendments or comparable ethical standards.

**Table 1:** Study population characteristics with a degree of hand focus, job, and hobbies

Groups	Subgroups	Participants (*N*)*		Mediator analysis (subgroups correlated to sex)
		Male	Female	
		38 (59%)	26 (41%)	*p* value
Age	20–30	12 (31.6%)	13 (50%)	> 0.05
	31–40	18 (47.4%)	5 (19.2%)	
	41–50	5 (13.2%)	4 (15.4%)	
	51–60	3 (7.9%)	4 (15.4%)	
Handedness	Right	37 (97.4%)	25 (96.2%)	
	Left	0 (0%)	1 (3.8%)	
	Both	1 (2.6%)	0 (0%)	
Job	Medical staff	24 (63.2%)	13 (50%)	
	Handwork	1 (2.6%)	1 (3.8%)	
	Administration/IT	13 (34.2%)	12 (46.2%)	
	Handicraft [hours/day]	2.1 ± 2.1	2.1 ± 2.2	
Playing an instrument	No	16 (42.1%)	15 (57.7%)	
	Yes	22 (57.9%)	11 (42.3%)	
	Degree of hand focus**	9.4 ± 1.1	9.6 ± 0.9	
Frequent sports activity	No	8 (21.1%)	2 (7.7%)	
	Yes	30 (78.9%)	24 (92.3%)	
	Degree of hand focus**	5.5 ± 1.3	5.6 ± 0.7	
Degree of integration of sports in everyday life	Rare/not at all	5 (13.2%)	2 (7.7%)	
	Less than half of all opportunities	6 (15.8%)	5 (19.2%)	
	More than half of all opportunities	8 (21.1%)	6 (23.1%)	
	Often/always	18 (47.4%)	13 (50%)	
Other hobbies	No	19 (50%)	13 (50%)	
	Yes	19 (50%)	13 (50%)	
	Degree of hand focus**	9.1 ± 1.8	9.0 ± 2.0	

^*^ diverse (*n *= 0)

^**^ Degree of hand focus: 0 = foot focused; 6 = equal; 11 = hand focused

### Recruitment and training of participants

Recruitment, training, and information provided to the volunteers were standardized and thus carried out by the same staff (male MD student). All of the 64 participants underwent a standardized training session prior to performing the simulated endovascular procedures by themselves. The training session included watching a standardized training video, in which basic interventional techniques were slowly demonstrated and explained by a board-certified interventionalist. Afterwards, participants were introduced to the silicone vascular model and the basic handling of each catheter was demonstrated once more in a standardized way. We did not aim at providing perfect training for every subject, but to provide a comparable training level giving all participants an approximately similar chance to solve the tasks. The intensity of training, as well as the simulated endovascular tasks, were set to reveal differences between the subjects. Several preliminary experiments with different volunteers were performed in order to optimize our study setup prior to starting the underlying study. None of the volunteers who were used to adjust the setup and difficulty level of the study was included in the real study. Finally, we created a set of four tasks with increasing and decreasing difficulty levels. The third task (task 2.1) was the most difficult task, whereby the first and the fourth task were the easiest.

### Catheter model and interventional tasks

**Fig. 1:**
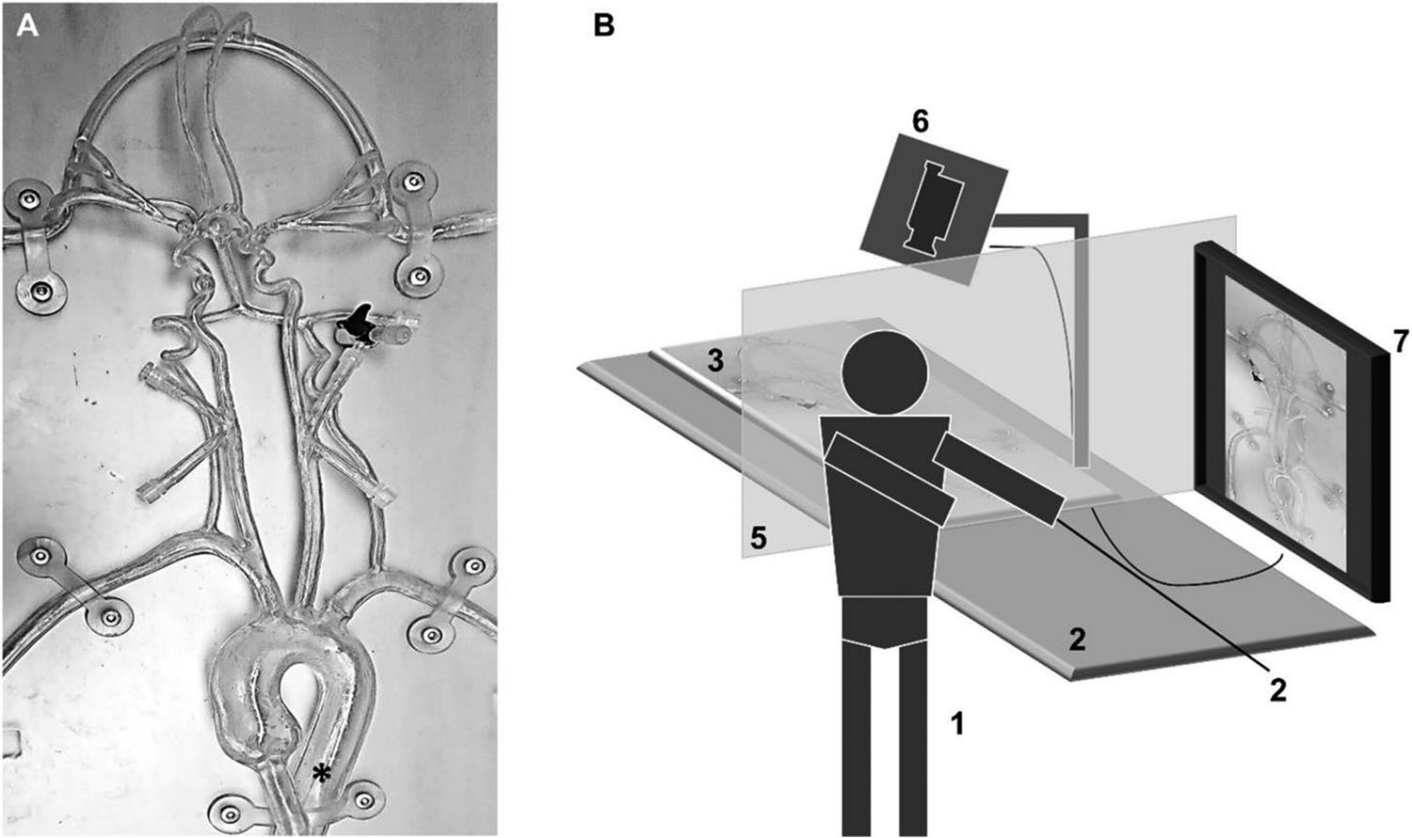
Experimental setup. **A** Photograph of the silicone vessel model used. All participants worked with the same vessel model to solve the given tasks with the starting position of the catheter (*) in the descending upper thoracic aorta. **B** Experimental setup: Participants (1) were standing at an angiography stretcher table (2) on which the silicone-vessel-model (3) was placed. The catheter system (4) was already in place at the starting position within the silicone model. To more realistically simulate angiography, the participants’ sight onto the silicone model was blocked by a curtain (5). A video camera (6) mounted over the silicone model was used to capture all catheter movements while simultaneously providing live feedback for the participants on a 32”-monitor (7) placed in front of the interventionalists

A life-size silicone model (NST00V02 #5117; United Biologics Inc.), ranging from the femoral artery up to the superior sagittal sinus, was used for the experiments (Fig. 1A). The silicone model was filled with distilled water mixed with baby shampoo (100:1). Each participant started the procedure from the same catheter position in the upper descending Aorta (marked with an asterisk in Fig. 1A). The complete experimental setup is shown in Fig. 1B.

**Fig. 2:**
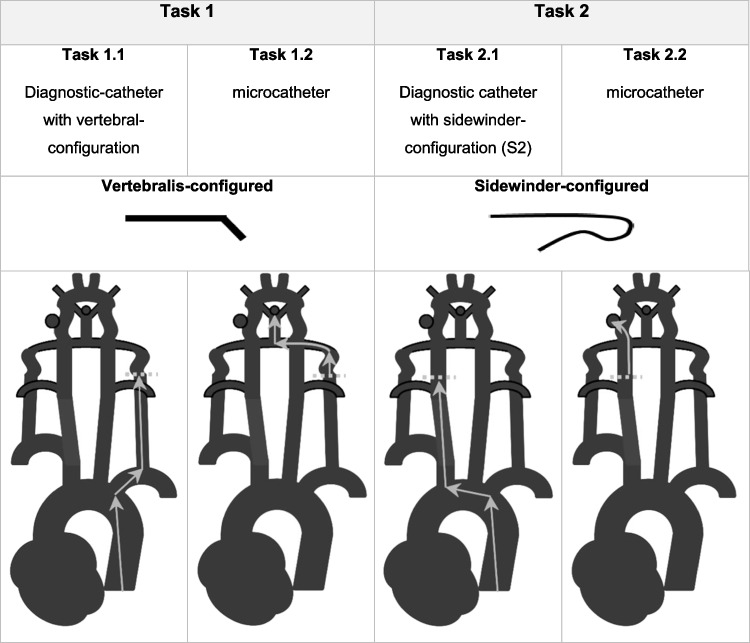
Interventional tasks. The goal of experiment 1.1 was to probe the left vertebral artery (arrows) using a vertebral-configured catheter. Task 1.2 continued where task 1.1 ended: The goal here was to probe a basilar-tip-aneurysm with a microcatheter via a microwire. Goal of experiment 2.1 was to probe the right internal carotid artery using a sidewinder-configured (Simmons 2) catheter. After completing task 2.1, goal of task 2.2 was to probe an aneurysm of the right internal carotid artery using a microcatheter and microwire

The participants were asked to solve four different tasks simulating neuroradiological interventional procedures (Fig. 2). The first task included using a vertebral-configured 4F-catheter to probe the V2-segment of the left vertebral artery (VA; Task 1.1), and from this point a microcatheter and a microwire had to be used to navigate the tip of the microcatheter into a tip-aneurysm of the basilar artery (BA; task 1.2). In the next task (2.1), an S2-configured 5F-catheter had to be used to probe the right internal carotid artery (ICA), from where a microcatheter and a microwire had to be used to navigate the tip of the microwire into an ICA-sidewall aneurysm.

Directly after accomplishing all four tasks, the participants were asked to fill in a questionnaire including the NASA-Task Load Index (described in detail below).

### Objective study parameters

The following parameters were acquired for each participant and each task: Time required to solve each task (in seconds); distance covered by the catheter within the vascular model during each task (in centimeters; measurements were performed using the freely available software „Viana.net“ (*Free Software Foundation Inc.*); the number of movements within predefined areas of interest (= “AOI”) and a number of tries—one “try” was defined as a forward movement, followed by a correcting backward movement within an AOI was classified as one “try.” From the aforementioned parameters catheters’ velocity (pathway covered by catheter per time needed to solve a task), agitation (number of movements divided by time), and acceleration were calculated. Acceleration was calculated using the parameters time, pathway, and number of tries by using the formula below:

$$Acceleration=\frac{Velocity\;\lbrack\frac{cm}s\rbrack}{Number\;of\;Tries}=\frac{Pathway\;\lbrack cm\rbrack}{Time\;taken\;\left[s\right]\ast Number\;of\;Tries}$$

### Subjective study parameters

Subjective parameters included self-assessed performance, self-assessed stress level, perceived experiments’ difficulty of each participant, as well as the time until asking for help. Parameters of self-assessment were taken from the NASA-TLX (1–20 points) and were queried using a questionnaire comparable to other studies [15–19].

During the experiment, all participants were allowed to ask for assistance if required, albeit knowing that any assistance would be recorded. Assistance consisted of standardized phrases provided by the staff.

### Statistical analysis

The condition of normal distribution for the independent t-test was not fulfilled as calculated with the Shapiro-Wilk-Test. Dunnett’s multi-comparison test was performed to compare gender-based objective parameters mean values (2-sided; α = 0.05). Non-parametric Kruskal-Wallis test was used to re-evaluate significance levels between mean values (there were no relevant changes). Correlations between objectively measured parameters and subjective stress levels, and self-assessed difficulty and performance were analyzed using a Spearman-Test (partial ordinal scaled parameters). Mediation analysis showed no relevant interaction between subgroups [20] and no relevant interactions between the subgroups were detected. Multicollinearity was considered if the correlation was *r *>0.9 (*p *< 0.05) [21, 22]. All analyses were performed using the statistical software SPSS (Version 29, IBM) and the PROCESS-Macro for SPSS from Hayes [23] to exclude possible mediator-based effects within subgroups (see Table 1).

## Results

Data from 64 participants (26 female and 38 male) were acquired. We created a set of four tasks (1.1, 1.2, 2.1, and 2.2) with increasing and decreasing difficulty levels. The third task (task 2.1) was the most difficult one, whereby the first and the fourth task were the easiest, as indicated by the times required to solve the tasks. Thus, the most significant differences were revealed while absolving task 2.1. One female participant decided to quit during task 2.1 due to high stress levels. Data of this participant were only considered for experiments 1.1 and 1.2, as well as for the NASA-TLX (*n *= 64), but not for analysis of the overall performance (*n *= 63).

### Objective study parameters

In total, women took themselves (or required) significantly more time to solve all four tasks (*p *= 0.02; see Table 2). This effect was most evident in the third task 2.1 (navigating the tip of a sidewinder-catheter from the aortic arch into the brachiocephalic trunk), which turned out to be the most challenging task as male and female participants required significantly more time to solve this task than for any other task (*p *< 0.001 compared to all other tasks). Female participants carried out significantly less catheter movements per second (“agitation”) during the third task (*p *= 0.005) and showed a lower catheter velocity than men (*p *= 0.016).

**Table 2:** Objective parameter results

	Sex	Task 1.1		Task 1.2		Task 2.1		Task 2.2		Overall	
		Mean ± 1SD	*p*	Mean ± 1SD	*p*	Mean ± 1SD	*p*	Mean±1SD	*p*	Mean±1SD	*p*
Time to solve tasks [s]	M	52.7 ± 31.3	0.4	138 ± 65.5	0.3	233 ± 163	0.02	78.1 ± 74.8	0.1	502 ± 230	0.02
	F	59.1 ± 30.8		162 ± 100		357 ± 264		115 ± 101		689 ± 364	
Number of movements	M	286 ± 143	0.6	172 ± 98	0.1	1309 ± 807	0.4	78.7 ± 63.4	0.008	1845 ± 866	0.2
	F	310 ± 205		228 ± 164		1508 ± 968		144 ± 124		2170 ± 1095	
Agitation [movements/s]	M	6.2 ± 2.7	0.2	1.3 ± 0.6	0.4	6.0 ± 1.8	0.005	1.2 ± 0.8	0.02	14.8 ± 4.6	0.2
	F	5.4 ± 2.2		1.5 ± 0.7		4.8 ± 1.6		1.7 ± 1.0		13.3 ± 4.3	
Number of tries	M	5.6 ± 3.4	0.8	14.6 ± 8.5	0.4	25.7 ± 13.9	0.1	9.8 ± 10.0	0.1	55.6 ± 23.3	0.056
	F	5.8 ± 3.9		16.7 ± 12.4		34.2 ± 27.4		14.2 ± 12.5		70.9 ± 39.0	
Catheter-tip-pathway [cm]	M	26.4 ± 10.1	0.3	30.4 ± 12.8	0.3	147 ± 90.2	0.2	17.0 ± 13.8	0.06	220 ± 100	0.07
	F	29.6 ± 16.9		35.0 ± 20.1		188 ± 123		24.7 ± 18.2		276 ± 135	
Velocity [cm/s]	M	0.6 ± 0.21	0.4	0.25 ± 0.07	0.8	0.66 ± 0.17	0.016	0.24 ± 0.1	0.1	1.76 ± 0.45	0.4
	F	0.54 ± 0.16		0.24 ± 0.1		0.57 ± 0.16		0.29 ± 0.13		1.66 ± 0.44	
Acceleration [cm/(s*try)]	M	0.1 ± 0.1	0.4	0.02 ± 0.02	0.6	0.04 ± 0.04	0.7	0.05 ± 0.1	0.1	0.3 ± 0.1	0.6
	F	0.1 ± 0.1		0.02 ± 0.02		0.04 ± 0.04		0.08 ± 0.1		0.3 ± 0.2	

Task 1.1 (probing the left VA with a vertebral catheter) was easiest and completed within the shortest time by all participants. For time, statistical significance was only reached in task 2.1 (probing the right ICA; *p *= 0.02). Acceleration of the catheter, number of tries, and catheter pathway did not show any significant differences between male and female participants.

Testing for multicollinearity in men and women, there were correlations between the number of movements and catheter pathway (each *r *> 0.9; *p *< 0.001), which described redundant effects.

### Subjective study parameters

Women rated their performance significantly lower than male participants (9.1 ± 3.3 vs. 11.3 ± 3.3; *p *= 0.009; Table 3). Concordantly, the difficulty of the experiments was rated significantly higher by females (11.5 ± 4.2 vs. 9.6 ± 3.3; *p *= 0.016), and they also reported significantly higher self-assessed stress levels during the tasks (8.9 ± 4.9 vs. 6.3 ± 4.4; *p *= 0.037; CI 95%; Table 3). In the first set of experiments (1.1 and 1.2) men (*n *= 5) asked for help earlier (after 130 ± 37s vs. 170 ± 138s; *p *= 0.6; 95% CI) than women (*n *= 8; Table 3). No significant differences were notable, as the first two tasks did not seem too difficult (as indicated before). In the more difficult second set of tasks (2.1 and 2.2), more women asked for help (19/25; 76% vs. 8/38; 21%) and did so significantly earlier (in Table 3 after 204 ± 95s vs. 305 ± 142s *p *= 0.049; 95% CI).

**Table 3:** Subjective parameter results

	Tasks 1.1 and 1.2			Tasks 2.1 and 2.2		
	Sex (*N*)	Mean ± 1 SD	*p* value	Sex (*N*)	Mean ± 1 SD	*p* value
Time until requesting help [s]	M (5)	130 ± 37	0.6	M (8)	305 ± 142	0.049
	F (8)	170 ± 138		F (19)	204 ± 95	
Number of supports per person	M (5)	2.8 ± 3.5	0.2	M (8)	4.4 ± 3.2	0.8
	F (8)	3.3 ± 1.7		F (19)	4.6 ± 2.7	
	After performing all tasks					
	Sex (*N*)	Mean ± 1 SD				*p* value
Self-assessed performance	M (38)	11.3 ± 3.3				0.009
	F (26)	9.1 ± 3.3				
Self-assessed difficulty	M (38)	9.6 ± 3.3				0.016
	F (26)	11.5 ± 4.2				
Self-assessed stress level	M (38)	6.3 ± 4.4				0.037
	F (26)	8.9 ± 4.9				

The time until asking for help increased significantly (*p *< 0.001) between the first (1.1/1.2) and the last two tasks (2.1/2.2), which could be due to a learning effect and increased motivation. Interestingly, the time until asking for help between the first and the second set of tasks increased stronger in men (+134%), whereas this increase was smaller in women (+20%; Table 3).

### Correlation between objective and subjective parameters

In general, female participants were able to correlate their objective performance and the perceived level of difficulty much better, as indicated by multiple significant correlations in Table 4. In contrast, correlations of objective performance parameters with self-assessed performance and difficulty failed to reach statistical significance in male participants.

**Table 4:** Correlation analysis results using Spearman’s rank correlation

		Self-assessed performance		Self-assessed difficulty		Self-assessed stress level	
		m	f	m	f	m	f
Time to solve tasks [s]	*R*	−.057	−.513	.188	.459	.469	.249
	*p* value	.732	.009	.258	.021	.003	.230
Number of movements	*R*	−.159	−.555	.068	.037	.587	.038
	*p* value	.339	.004	.687	.861	< 0.001	.858
Agitation [movements/s]	*R*	.103	−.053	−.256	−.410	−.072	–.251
	*p* value	.538	.803	.120	.042	.666	.226
Number of tries	R	.054	−.394	.187	.379	.399	.142
	*p* value	.756	.051	.262	.061	.013	.498
Catheter-tip-pathway [cm]	*R*	−.113	−.469	.135	.179	.459	.167
	*p* value	.498	.018	.420	.391	.004	.424
Velocity [cm/s]	*R*	.044	−.005	−.211	−.266	−.110	–.131
	*p* value	.795	.981	.203	.199	.511	.532
Acceleration [cm/s*try]	*R*	.125	.383	−.182	−.469	−.233	–.429
	*p* value	.455	.059	.275	.018	.159	.033
*N*		38	25	38	25	38	25

On the other hand, perceived stress levels correlated significantly with most of the objectively assessed parameters in male participants (*p* between < 0.001 and 0.013; Table 4), but interestingly not in female participants for 6 out of 7 parameters (except catheter-acceleration correlating with stress level in women; here *p *= 0.033).

## Discussion

The number of studies analyzing “gender differences” increased exponentially within the last three decades, as confirmed by a PubMed search. The same applies to the search terms gender differences and medicine. Focusing on medicine, significant gender differences in medical treatment and outcomes have been described for innumerous diseases [1, 2, 4–6, 13]. Likewise, significant differences between male and female physicians affecting medical training as well as daily clinical practice have been identified [3, 8, 10, 14]. In the underlying cohort study not only technical skills but even more important factors such as perceived stress levels and the ability to assess one self’s performance correctly were analyzed.

Basic objective parameters, such as time and a number of catheter movements, were the most primitive and superficial sort of parameters, which we acquired by analysing the procedures. From these data, we additionally calculated further parameters such as velocity and agitation. Our experimental setup contained four different tasks with different difficulty levels. The most challenging task was task 2.1, which significantly (by about 40–55%) contributed to the overall results of the objective parameters (because the participants took more time to manage this task). Whereas there were no significant differences in objective parameters in the easier tasks, we observed that the female participants took more time to solve the given tasks and asked earlier for help than the male participants. On the other hand, women were able to estimate their own objective performance much better, whereas men failed to correctly assess their own performance. The difference in working speed might result from different working strategies and it could also be triggered by different factors. For example, all participants were informed that the time was taken during the experiments. However, it was never said to any of the participants that time is of relevance to judge their performance. Nevertheless, this information alone could be sufficient to trigger male competitiveness (which from an evolutionary aspect may be expected to have been exposed to strong selective pressure throughout human history) [24]. Therefore, it could have been that male participants simply hurried up because they felt the experiment to be a challenge, while women did not see this experiment as a challenge and simply took their time to solve the tasks. This would be in line with the hypothesis, that reduced working speeds yield a higher degree of precision thereby avoiding more complications and resulting in a better outcome. This interpretation would also be supported by the findings of Barr et al, who reported women take more time in treating patients [1, 2, 4], which resulted in an increased level of patient satisfaction [1, 5]. Gender-based treatment differences were also observed in terms of 30-day mortality and readmission in an internists’ study: patients treated by female internists presented a significantly lower readmission rate and mortality after 30 days than patients treated by male internists [6]. Furthermore, Alcaide-Leon et al observed fewer mistakes made by female doctors in diagnostic radiology than by their male colleagues [25]. Irrespective of disease severity, patient-specific characteristics, and gender, treatment disparities have also been related to a surgeon’s age [26]. This, however, is not an unexpected finding as older surgeons in most cases will be more experienced.

Accordingly, gender differences in self-assessment and perception of one’s own abilities and achievements have been reported. Women, for example, do not only seem to rate their performance generally lower than men [7] but women and men also seem to perceive personal success and failure differently [27, 28]. Whereas women tend to rate their personal success as “*lucky*”, and failures are considered as “*lack of skills”*, men tend to rate their personal success as “*based on skill”* and failures as *being “unlucky”* [27, 28]. Interestingly, when being unobserved, women rated their performance similar to those of their male counterparts [7]. Ludwig et al thus hypothesized that women, when being under observation, seem to judge their own performance more accurately than men, presumably to avoid being judged negatively for their failings [7]. This explanation would also match our observation with women requiring more time to solve the interventional tasks in our experimental setup while correctly interpreting their own performance. These findings are also supported by Gill et al, who exposed male and female participants to tasks harbouring a high likelihood of failure [3]: They observed a decreased performance of male participants concomitantly with a decreased chance of success, whereas female performance decreased independently of that. In our study, higher stress levels of male participants positively correlated with an increase in time and number of catheter movements required to solve the tasks. Thus, when men were more stressed out, they tended to become more agitated. When women indicated lower stress levels and lower difficulty of tasks and self-assessed their performance higher, they showed higher catheter acceleration values than men, which could mean, that these female participants performed catheter movements more efficiently than men.

The present study has some limitations. Whereas over 60 volunteers were recruited, the number of participants still could have been higher. On the other hand, the number of participants yielded several significant results. We tried to eliminate any bias from the study using a standardized teaching video and recruitment, training, and the experiments were performed by the same male MD student. This, in fact, might have introduced some bias, as male or female participants might have reacted differently depending on the gender (or even personality) of the study supervisor. This problem, however, is almost intractable. Interestingly, we realized that men were more willing to participate in the study than women, which is another potential source for bias, which hardly can be corrected. Reasons for this might be that men are more prone to accept challenges. This theory would also be supported by Ludwig et al and Gill et al, who reported a higher willingness of male participants to accept challenges and to compare with others than women. Ludwig et al hypothesized that women could tend to rate their performance lower when being observed, which would match our results. Whether this difference affects the results of our study finally remains unclear, and matched-pair analyses of a larger cohort might yield even more exact results. The fact that we investigated the performance of volunteers naïve to catheter techniques puts the results of this study mainly into an early educational/training setting: comparing well-trained (and it would be difficult to clearly define “well-trained”) male and female interventionalists might have yielded different results. Therefore, performing this experiment with participants naïve to catheter handling provides the considerable advantage of avoiding confounding factors such as years of training. Another important point is that differences in objective catheter handling performance might have influenced the results. Whereas we ensured that none of the participants had any experience with catheter handling, it was more difficult to exclude different backgrounds regarding other situations in which hand-eye coordination is important. We therefore checked the background of the participants using a questionnaire including activities possibly affecting manual dexterity including sports and playing an instrument but found no significant differences here.

Differences in self-assessed performance, stress level, and self-assessed difficulty observed in our study were comparable to the results of other studies [3, 7, 27, 28] and could explain differences in performance between men and women. Increased awareness of failing in women might already have influenced our objective parameters, such as perceived stress levels, difficulty, and own performance. One consequently may interpret that the female participants presented with a remarkably critical self-perception, which may have resulted in a raised willingness to ask for help. Most interestingly, when our male participants felt their skills were insufficient, their self-assessed stress levels increased proportionally.

In conclusion, we observed several interesting gender-related differences not only regarding working speed and catheter movements in some parts of the experiment but also in self-perception of individual performance and stress levels in this educational setting. We learned that inexperienced male volunteers solved the most difficult interventional task somewhat faster than women, who in contrast took themselves more time and performed catheter movements with less agitation. Female participants asked more frequently and earlier for help than their male counterparts, while they showed a more “aggressive tactic” with significantly more catheter movements per second. Female participants inexperienced in endovascular procedures rated their performance in general lower than inexperienced men and indicated higher stress levels during simulated angiography, but were much better able to correctly assess their objective performance. Whereas men tended to overestimate their own performance and failed in self-assessment of objective parameters. Although these gender-based differences do not necessarily reflect the situation in well-trained male and female interventionalists, instructors should be aware of these differences as they may affect the interpretation of young colleagues’ behavior and performance, thereby improving training and teaching of not only interventionalists.

## Abbreviations

AOI: Area of interest
BA: Basilar artery
ICA: Internal carotid artery
IR: Interventional radiology
NASA-TLX: National Aeronautics and Space Administration-Task Load Index
VA: Vertebral artery

## Declarations

### Guarantor

The scientific guarantor of this publication is Marc A. Brockmann.

### Statistics and biometry

AR and MAB kindly provided statistical advice for this manuscript.

### Methodology

• Prospective

• Experimental

• Performed at one institution

## References

[1] Barr DA (2017). Gender Differences in Medicine-From Medical School to Medicare. Mayo Clin Proc.

[2] Barr DA, Vergun P, Barley SR (2000). Problems in using patient satisfaction data to assess the quality of care provided by primary care physicians. J Clin Outcomes Manag.

[3] Gill D, Prowse V (2014). Gender differences and dynamics in competition: the role of luck. Quant Econ.

[4] Roter DL, Hall JA, Aoki Y (2002). Physician gender effects in medical communication: a meta-analytic review. JAMA.

[5] Cooper-Patrick L, Gallo JJ, Gonzales JJ (1999). Race, gender, and partnership in the patient-physician relationship. JAMA.

[6] Tsugawa Y, Jena AB, Figueroa JF, Orav EJ, Blumenthal DM, Jha AK (2017). Comparison of hospital mortality and readmission rates for Medicare patients treated by male vs female physicians. JAMA Intern Med.

[7] Ludwig S, Fellner-Röhling G, Thoma C (2017). Do women have more shame than men? An experiment on self-assessment and the shame of overestimating oneself. Europ Econ Rev.

[8] Molwitz I, Yamamura J, Ozga A-K (2021). Gender trends in authorships and publication impact in Academic Radiology—a 10-year perspective. Eur Radiol.

[9] Brotherton SE, Etzel SI (2014). Graduate Medical Education, 2013–2014. JAMA.

[10] Rosenkrantz AB, Englander MJ, Deipolyi AR, Findeiss L, Duszak R (2019). Clinical practice patterns of interventional radiologists by gender. Am J Roentgenol.

[11] Bedoya-Vaca R, Derose KP, Romero-Sandoval N (2016). Gender and physician specialization and practice settings in Ecuador: a qualitative study. BMC Health Serv Res.

[12] Asaad M, Zayegh O, Badawi J (2020). Gender differences in specialty preference among medical Students at Aleppo University: a cross-sectional study. BMC Med Educ.

[13] Kubik-Huch RA, Vilgrain V, Krestin GP (2020). Women in radiology: gender diversity is not a metric—it is a tool for excellence. Eur Radiol.

[14] Li O, Ross M, Wiseman D (2021). Women in Interventional Radiology: exploring the Gender Disparity in Canada. Curr Probl Diagn Radiol.

[15] Grier RA (2015) How high is high? A meta-analysis of NASA-TLX global workload scores Proc Hum Factors Ergon Soc Annu Meet. SAGE, pp 1727-1731

[16] Hart SG (2006) NASA-task load index (NASA-TLX); 20 years laterProc Hum Factors Ergon Soc Annu Meet. SAGE, pp 904-908

[17] Hart SG (1986) NASA Task load Index (TLX). Volume 1.0; Paper and pencil package

[18] Law KE, Lowndes BR, Kelley SR (2020). NASA-task load index differentiates surgical approach: opportunities for improvement in colon and rectal surgery. Ann Surg.

[19] Lowndes BR, Forsyth KL, Blocker RC (2020). NASA-TLX Assessment of Surgeon Workload Variation Across Specialties. Ann Surg.

[20] Baron RM, Kenny DA (1986). The moderator–mediator variable distinction in social psychological research: conceptual, strategic, and statistical considerations. JPSP.

[21] Verma J (2015) Repeated measures design for empirical researchers. John Wiley & Sons, pp 191

[22] Tabachnick BG, Fidell LS (2013). Using multivariate statistics.

[23] Hayes AF, Montoya AK, Rockwood NJ (2017). The analysis of mechanisms and their contingencies: PROCESS versus structural equation modeling. AMJ.

[24] Alger I (2021). On the evolution of male competitiveness. J Econ Behav Organ.

[25] Alcaide-Leon P, Rawal S, Krings T (2022). Gender differences in diagnostic radiology practice: an observational study. J Am Coll Radiol.

[26] Tsugawa Y, Jena AB, Orav EJ et al (2018) Age and sex of surgeons and mortality of older surgical patients: observational study. bmj 361 10.1136/bmj.k1343 PMC5915700 29695473

[27] Minter RM, Gruppen LD, Napolitano KS, Gauger PG (2005). Gender differences in the self-assessment of surgical residents. Am J Surg.

[28] Beyer S, Bowden EM (1997). Gender differences in seff-perceptions: convergent evidence from three measures of accuracy and bias. Pers Soc Psychol Bull.

